# *Clinical Epigenetics *is now a fully open access journal

**DOI:** 10.1186/1868-7083-3-1

**Published:** 2011-10-26

**Authors:** Ulrich Mahlknecht

**Affiliations:** 1 Saarland University Medical Center, Department of Internal Medicine, José Carreras Research Center, D-66421 Homburg/Saar, Germany

## Introductory Editorial

We are pleased to announce this first open access volume of *Clinical Epigenetics *following its transfer to BioMed Central one year after appearance of the first article in *Clinical Epigenetics *[1].

*Clinical Epigenetics *is a peer-reviewed journal, which publishes original work of exceptional quality and interest, providing a wide-ranging coverage of research, views, and reviews on epigenetic principles and mechanisms, and their effects in relation to human health and disease. *Clinical Epigenetics *includes epigenetic research in humans and various disease model organisms, is of interest to the basic researcher as well as to the physician and clinician. The journal aims to provide a forum for those interested in practical questions relating to clinical epigenetics, and the implications of epigenetics on human health and disease. *Clinical Epigenetics *is the official publication of the Clinical Epigenetics Society [2], a non-profit international association of physicians, scientists, and interested individuals with the goal of promoting and supporting scientific research and communication within the field of clinical epigenetics. *Clinical Epigenetics *is the first journal dedicated to the connection of epigenetic patterns to human physiology and disease.

The transition of *Clinical Epigenetics *to an open access publishing model has been made with the intention to reach out to the continuously increasing audience that expresses an interest in the field [3-6]. All articles will now be freely and universally accessible online to a much larger readership than any subscription-based journal [7]. While article-processing charges, in the form of a flat fee with no additional charges for colour figures, movies and large datasets, apply upon acceptance of a manuscript, all articles will be available to readers at no cost and will not be limited by their library's budget or personal subscriptions, ensuring that the work published in *Clinical Epigenetics *is disseminated within the widest possible audience [7].

The open access publishing model brings a number of considerable advantages to the journal including better indexing opportunities, immediate publication upon acceptance and high visibility within the field. In addition, we anticipate that the number of online downloads will continuously increase, as well as the number of citations, potentially leading to a high citation impact [8,9]. Authors hold the copyrights for their work and grant anyone the right to reproduce and disseminate the article, provided that it is correctly cited and no errors are introduced. In addition, the journal's articles are deposited in widely and internationally recognized open access repositories. This complies with the policies of a number of funding bodies including the Wellcome Trust, NIH and Howard Hughes Medical Institute [10-13]. Particularly in view of recurring worldwide financial and economic crises that may affect some countries more than others, it is important to us that the open access publishing model guarantees that a country's economy will not influence its researchers' ability to access articles because resource-poor countries (and institutions) will be able to read the same material as wealthier ones [14].

We are delighted to be taking *Clinical Epigenetics *forward with BioMed Central, and look forward to its success as an open access journal, providing an international forum for the latest epigenetics research relating to human health and disease.
